# THE MEDIATING ROLE OF PAIN SEVERITY IN THE ASSOCIATION BETWEEN DEPRESSIVE SYMPTOMS AND GAIT SPEED

**DOI:** 10.1093/geroni/igad104.2449

**Published:** 2023-12-21

**Authors:** Rhea Mehta, Michelle Shardell, Alice Ryan, Yu Dong, Brock Beamer, Marc Hochberg, Alan Rathbun

**Affiliations:** University of Maryland Baltimore, Baltimore, Maryland, United States; University of Maryland School of Medicine, Baltimore, Maryland, United States; University of Maryland, Baltimore, Baltimore, Maryland, United States; University of Maryland, Baltimore, Baltimore, Maryland, United States; University of Maryland, Baltimore, Baltimore, Maryland, United States; University of Maryland, Baltimore, Baltimore, Maryland, United States; University of Maryland, Baltimore, Baltimore, Maryland, United States

## Abstract

Knee osteoarthritis (OA) and comorbid depression adversely impact gait speed over time. Depression can alter pain tolerance and perception; therefore, self-reported knee pain severity may mediate the relationship between depressive symptoms and gait speed, but this has not been evaluated longitudinally using repeated measures data. Thus, we assessed whether knee pain severity mediates the association between depressive symptoms and gait speed over time. Participants (n=2,222) from the Osteoarthritis Initiative with radiographic knee OA (Kellgren-Lawrence grade ≥ 2) in at least one knee were included. Depressive symptoms were assessed using the Center for Epidemiologic Studies Depression Scale (CES-D; range 0-60) at baseline and first two annual follow-up visits. The twenty-meter gait speed (meters per second) outcome was assessed at the second through fourth annual follow-up visits. Knee pain severity was assessed as a mediator and measured using the Western Ontario and McMaster Universities Osteoarthritis (WOMAC) pain subscale at the first through third annual follow-up visits. Linear regression mediation using a version of the Sobel-Goodman approach was the primary method of analysis. Indirect and direct effects of depressive symptoms on gait speed for each one-unit CES-D score were -0.001 (p < 0.001) and -0.003 (p = 0.020) standard deviations, respectively. Thus, approximately 24% of the association between depressive symptoms and gait speed was mediated by knee pain severity across time. The current results support the need for simultaneous, multidisciplinary interventions for both depressive symptoms and knee OA pain to prevent future decline in physical performance.

